# Epicardial adipose tissue affects the efficacy of left atrial posterior wall isolation for persistent atrial fibrillation

**DOI:** 10.1002/joa3.12359

**Published:** 2020-05-16

**Authors:** Yosuke Nakatani, Tamotsu Sakamoto, Yoshiaki Yamaguchi, Yasushi Tsujino, Koichiro Kinugawa

**Affiliations:** ^1^ Second Department of Internal Medicine University of Toyama Toyama Japan

**Keywords:** atrial fibrillation, catheter ablation, epicardial adipose tissue, left atrium, posterior wall isolation

## Abstract

**Background:**

Epicardial adipose tissue (EAT) contributes to atrial fibrillation (AF). However, its impact on the efficacy of left atrial posterior wall isolation (LAPWI) is unclear.

**Methods:**

Forty‐four nonparoxysmal AF patients underwent LAPWI after pulmonary vein isolation. EAT overlap on LAPWI was assessed by fusing computed tomography images with electro‐anatomical mapping.

**Results:**

During the 21 ± 7 months of follow‐up, AF recurred in 10 patients (23%). The total and left atrial EAT volumes were 113 ± 36 and 33 ± 12 cm^3^, respectively. No differences were found between the AF‐free and AF‐recurrent groups regarding EAT volume. The EAT overlaps on LAPWI lines and LAPWI area were 1.2 ± 1.0 and 0.5 ± 0.9 cm^2^ respectively. Although no difference was found between groups regarding the EAT overlap on LAPWI area, the AF‐free group had a significantly larger EAT overlap on LAPWI lines (1.4 ± 1.0 vs 0.6 ± 0.6 cm^2^, *P* = .014). Multivariate analysis identified EAT overlap on LAPWI lines as an independent predictor of AF recurrence (hazard ratio: 0.399, 95% confidence interval: 0.178‐0.891, *P* = .025). Kaplan‐Meier analysis revealed that, during follow‐up, 92% of the large EAT overlap group (≥1.0 cm^2^) and 58% of the small EAT overlap group (<1.0 cm^2^) remained AF‐free (*P* = .008).

**Conclusions:**

EAT overlap on LAPWI lines is related to a high AF freedom rate. Direct radiofrequency application to EAT overlap may be necessary to suppress AF.

## Graphical abstract

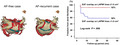

## 1 INTRODUCTION

Epicardial adipose tissue (EAT) is a metabolically active tissue located between the visceral pericardium and the epicardium, known as a risk factor for various cardiovascular diseases,^1^ particularly atrial fibrillation (AF), to which EAT is closely related. EAT volume is related to both new‐onset AF^2^, ^3^ and AF recurrence after catheter ablation^4^, ^5^ or cardioversion.^6^ Moreover, EAT alters the atrial electrophysiological properties contributing to generate AF.^7^, ^8^

EAT promotes AF both through local and remote effects. The local effect involves adipocyte infiltration into the atrial myocardium^9^, ^10^ and fibrosis of the neighboring atrial tissue by paracrine secretion of profibrotic adipokines.^11^, ^12^, ^13^ The remote effect, on the other hand, arises from the systemic secretion of adipokines and metabolites.^14^ Furthermore, ganglionated plexuses are located in EAT, altering the electrophysiological properties of atrial remote areas.^15^, ^16^ It remains unclear whether the main pathway underlying the contribution of EAT for the development of AF involves the local or remote effect.

Previous studies^17^, ^18^ have revealed that applying radiofrequency to the areas overlapped with EAT suppressed AF recurrence after catheter ablation. However, if the local effect is the main pathway for the AF‐promoting effect of EAT, isolation of the areas overlapping with EAT should be sufficient to suppress AF. If, on the other hand, the remote effect contributes to AF development, applying radiofrequency directly to the areas overlapped with EAT should be needed. Since left atrial posterior wall isolation (LAPWI) creates an isolated area in the left atrium, assessing EAT overlap on LAPWI may provide new insights into the mechanisms of the AF‐promoting effect of EAT. Therefore, the present study aimed to investigate the impact of EAT overlap on AF recurrence after catheter ablation, including LAPWI in patients with persistent AF.

## 2 METHODS

### 2.1 Study population

The present study retrospectively analyzed consecutive patients with persistent and long‐standing persistent AF who underwent catheter ablation at the Toyama University Hospital (Toyama, Japan) from November 2015 to October 2016. Persistent AF was defined as AF lasting ≥7 days but <1 year, whereas long‐standing persistent AF was defined as continuous AF lasting ≥1 year. Patients with previous ablation, prior heart surgery, thyroid diseases, renal dysfunction, and contrast allergy were excluded. Overall, 44 patients were enrolled (persistent AF, 26; long‐standing persistent AF, 18). Clinical characteristics were obtained from the patients’ medical records. The study protocol was approved by the Research and Ethics Committee of the University of Toyama (Toyama, Japan) and conducted in accordance with the principles of the Declaration of Helsinki. All patients provided written informed consent.

### 2.2 Catheter ablation and mapping

All antiarrhythmic drugs were discontinued for at least five half‐lives, and no patient received oral amiodarone prior to ablation. Sheath introducers were inserted through the right femoral vein under sedation. A transseptal procedure was performed, and two 8‐F SL0 sheaths and a steerable sheath (Agilis, St. Jude Medical, Inc, St. Paul, MN, USA) were advanced into the left atrium. Three‐dimensional (3D) atrial geometry was created on the NavX system (St. Jude Medical, Inc) using 7‐F decapolar circular catheters (Lasso, Biosense‐Webster, Inc, Diamond Bar, CA, USA; Libero, Japan Lifeline Co., Ltd., Tokyo, Japan). An irrigated tip radiofrequency catheter (Flexibility, St. Jude Medical, Inc) was used for the radiofrequency application. Pulmonary vein isolation (PVI) was performed under the guidance of two 7‐F decapolar circular catheters (Lasso and Libero) positioned at the ipsilateral pulmonary vein ostia.

Sequential contact mapping was performed following PVI during AF. A 7‐F decapolar circular catheter (Libero) was used for mapping. The contact of the mapping catheter was validated both by electrogram stability and by the distance to the atrial geometry surface and catheter motion, concordant with the cardiac silhouette on fluoroscopy. Analyses of complex fractionated atrial electrograms (CFAEs) were performed in 342 ± 288 points. Fractionation analyses were performed on the NavX system for CFAE mapping. CFAE mapping was performed with the following settings: peak‐to‐peak sensitivity, 0.04 mV; electrogram refractory period, 30 ms; electrogram width, <10 ms; electrogram segment length, 5 seconds.^17^ The fractionated index was defined as the average time interval between consecutive deflections over each recording period. CFAE was defined as an average fractionated index of ≤120 ms^19^ The voltage map was constructed from the voltage data obtained during contact mapping. Low voltage areas were delineated based on a bipolar voltage of <0.5 mV (determined as the maximum bipolar voltage of three consecutive AF beats, excluding QRS complexes from the window of interest).

Following contact mapping, LAPWI was performed by creating a left atrial roof line at the most cranial aspect and a floor line joining the most inferior margin of the PVI line. If the left atrial posterior wall was not isolated, additional radiofrequency applications were performed, targeting the earliest activation site on the isolation lines. The entrance block was confirmed by voltage mapping using a 3D mapping system. The exit block was confirmed after external cardioversion using high output pacing within the LAPWI lines. The procedure was completed with the creation of a block line on the mitral isthmus and the cavotricuspid isthmus. The low voltage areas and CFAE areas were not ablated. Each radiofrequency application was performed for 30‐50 seconds. The temperature and power were maintained at 42°C and 30 W, respectively; a maximum power of 25 W was used while delivering energy to the sites near the esophagus.

### 2.3 Multidetector enhanced computed tomography imaging

All patients underwent contrast‐enhanced multidetector computed tomography (CT), which was performed using either a dual source 128‐slice multidetector CT‐scanner (Somatom Definition AS+; Siemens Medical Solutions, Forchheim, Germany; 0.30 seconds gantry rotation time, 120 kV) or a 64‐slice multidetector CT‐scanner (Somatom Sensation Cardiac 64; Siemens Medical Solutions; 0.33 seconds gantry rotation time, 120 kV) within 3 days prior to ablation. Images were captured during a breath hold at full expiration using cardiac gating from the aortic arch's caudal aspect, through the cranial aspect of the left hemidiaphragm. The slice was 0.6‐mm thick, and a reconstructed series in systole (20%–40% of the R–R interval) was selected for analysis.

### 2.4 Epicardial adipose tissue analysis

**Figure 1:**
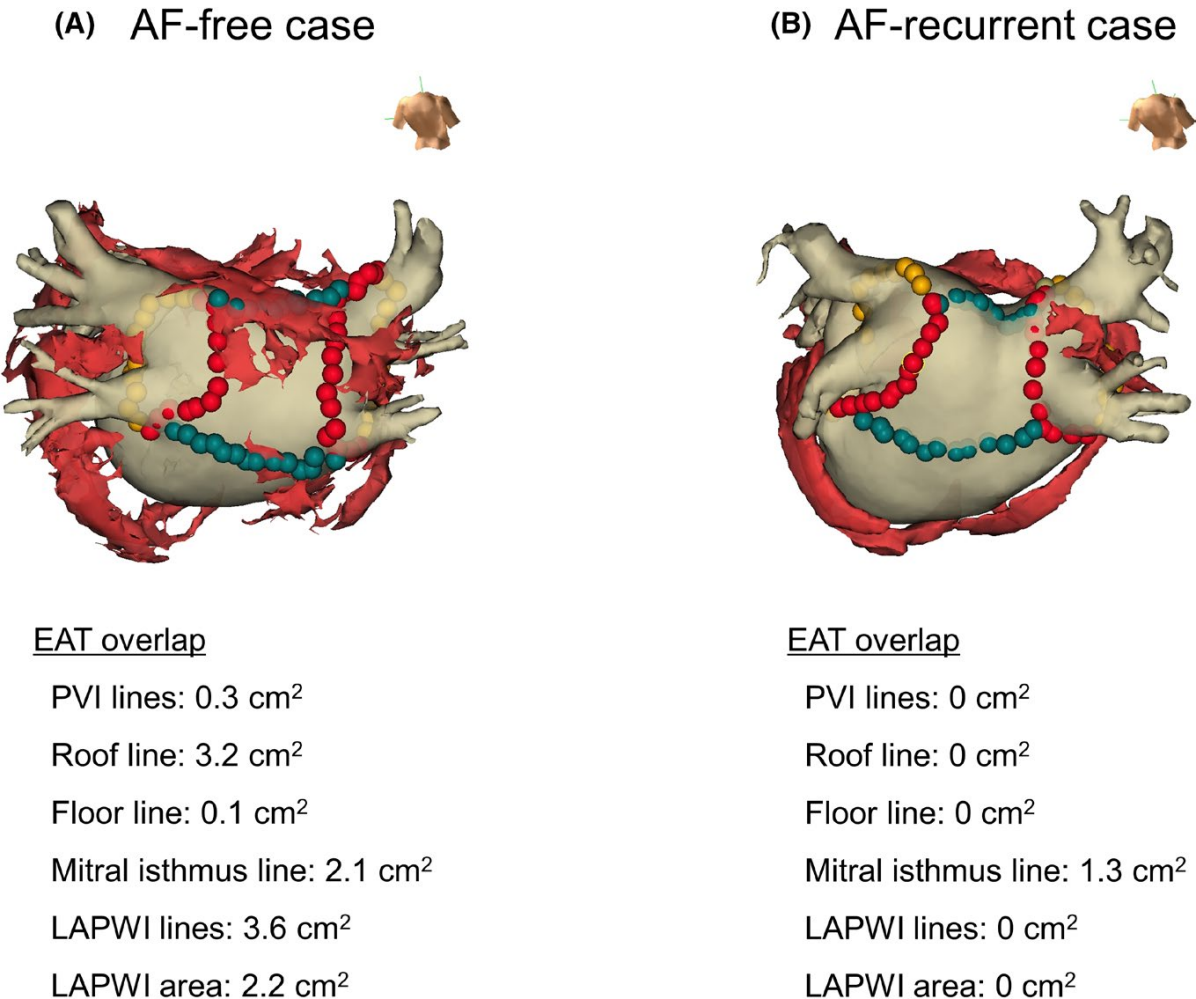
Representative cases of epicardial adipose tissue (EAT) overlap on ablation lines and the left atrial posterior wall isolation (LAPWI) area. Fusion of the computed tomographic image with EAT (red structures) and the geometry created on three‐dimensional mapping was performed. The red and green tags show the ablation points for pulmonary vein isolation (PVI) and LAPWI respectively. (A) Atrial fibrillation (AF)‐free case. The EAT overlap on LAPWI lines and LAPWI area were 3.6 and 2.2 cm^2^ respectively. (B) AF‐recurrent case. The EAT overlap on LAPWI lines and LAPWI area were both 0 cm^2^

EAT was retrospectively assessed using both visualization and an analysis software (Synapse Vincent; Fujifilm, Tokyo, Japan), detected by assigning Hounsfield units from −50 to −200,^17^ and reconstructed from 0.6‐mm slices of axial images. Total EAT was defined as EAT within the pericardial sac from the pulmonary artery bifurcation level to the diaphragm level. EAT located on the ventricular side of the mitral annulus and the tricuspid annulus was manually deleted from the total EAT. Thereafter, the left atrial EAT was determined by deleting EAT to the right side of the interatrial septum line. CT data were transferred into the NavX system. The left atrium, pulmonary veins, and EAT were reconstructed and segmented on the attached image integration software (Ensite Verisimo). The CT image was then fused with the electro‐anatomical mapping and EAT overlaps on ablation lines were evaluated by two independent blinded observers. Moreover, EAT overlaps on the LAPWI ablation lines (ie PVI lines, roof line, and floor line) and the LAPWI area were also evaluated. EAT overlap was manually measured by tracing the area where EAT overlapped with the ablation sites or the LAPWI area. Representative cases are shown in Figure 1.

### 2.5 Postprocedure care and follow‐up

All patients were subjected to a clinical interview and a surface 12‐lead electrocardiogram on the day after ablation and thereafter during the monthly visits to the outpatient clinic. Twenty‐four‐hour Holter monitoring was performed on the day following catheter ablation and as needed thereafter the follow‐up period. AF recurrence was identified both from the symptoms along with documentation of an AF episode lasting ≥30 seconds, on a surface 12‐lead electrocardiogram or Holter monitoring after a 3‐month blanking period from ablation. Antiarrhythmic drugs were resumed at the responsible physician's discretion.

### 2.6 Statistical analysis

Results are presented as the mean ± standard deviation and 95% confidence intervals. Continuous variables were compared using the unpaired Student's *t*‐test, whereas categorical variables were compared using the *χ* ^2^ test. Univariate and multivariate Cox proportional hazards regression analyses were performed to identify AF recurrence predictors. Related factors with a *P* < .100 in the univariate analysis were selected as independent variables for multivariate analysis. Upon observing a significant correlation between two variables, the variable with lower significance was excluded from the multivariate analysis to eliminate the multicollinearity. Correlations between parameters were analyzed using the Pearson's correlation coefficient. Receiver operating characteristic curve analyses were performed to determine the optimal parameter cut‐off values for AF recurrence prediction. The outcome of ablation over time was plotted using a Kaplan‐Meier survival curve and compared between parameter groups (below vs above cut‐off) by log‐rank test. A *P* < .050 (two‐tailed) was accepted as statistically significant for all tests.

## 3 RESULTS

### 3.1 Patients’ characteristics and catheter ablation

Patients’ characteristics are shown in Table 1. The mean age was 64 ± 10 years and 84% of the patients were male. The mean body mass index was 25 ± 3 kg/m^2^. The mean AF duration was 16 ± 21 months and 41% of the patients had long‐standing persistent AF. Structural heart disease and congestive heart failure were observed in 32% and 20% of patients respectively. Prior to ablation, antiarrhythmic drugs were administered in only 11% of the patients. The left atrium was enlarged, and the left atrial appendage flow velocity was below the normal range. The left ventricular ejection function was preserved and the total and left atrial EAT volumes were 113 ± 36 and 33 ± 12 cm^3^ respectively.

**Table 1:** Patients’ characteristics

	All patients (n = 44)	AF‐free group (n = 34)	AF‐recurrent group (n = 10)	*P* value
Age, years	64 ± 10	65 ± 9	61 ± 13	.317
Male gender	37 (84)	30 (88)	7 (70)	.166
Body mass index, kg/m^2^	25 ± 3	25 ± 3	24 ± 3	.285
AF duration, months	16 ± 21	17 ± 23	12 ± 10	.545
Long‐standing persistent AF	18 (41)	13 (38)	5 (50)	.551
Structural heart disease	14 (32)	10 (29)	4 (40)	.527
Congestive heart failure	9 (20)	7 (21)	2 (20)	.968
Hypertension	23 (52)	20 (59)	3 (30)	.109
Diabetes mellitus	3 (7)	3 (9)	0 (0)	.331
Past history of stroke	6 (14)	6 (18)	0 (0)	.153
Antiarrhythmic drugs before ablation	5 (11)	5 (15)	0 (0)	.198
Antiarrhythmic drugs after ablation	22 (50)	16 (47)	6 (60)	.454
Left atrial volume, cm^3^	155 ± 32	158 ± 31	145 ± 31	.248
Left atrial appendage flow velocity, cm/s	38 ± 21	39 ± 22	35 ± 14	.639
Left ventricular ejection fraction, %	63 ± 8	64 ± 8	59 ± 8	.094
Total EAT volume, cm^3^	113 ± 36	116 ± 34	103 ± 43	.322
Left atrial EAT volume, cm^3^	33 ± 12	33 ± 13	33 ± 11	.922

Note: Data are mean ± SD or number (%) of patients.

Abbreviations: AF, atrial fibrillation; EAT, epicardial adipose tissue.

LAPWI was successfully performed in 44 patients (100%). Mitral isthmus block and cavotricuspid isthmus block were achieved in 40 patients (91%) and 44 patients (100%) respectively. Catheter ablation data are shown in Table 2. The left atrial surface area measured on 3D mapping was 106 ± 18 cm^2^, and the LAPWI area was 11 ± 4 cm^2^. The total radiofrequency time was 66 ± 16 minutes, and the radiofrequency application time needed for LAPWI was 8 ± 5 minutes. The low voltage areas observed in the left atrium and posterior wall were 57 ± 21 and 10 ± 5 cm^2^ respectively. The CFAE areas observed in the left atrium and posterior wall were 31 ± 17 and 2 ± 3 cm^2^ respectively.

**Table 2:** Catheter ablation data

	All patients (n = 44)	AF‐free group (n = 34)	AF‐recurrent group (n = 10)	*P* value
Left atrial surface area, cm^2^	106 ± 18	107 ± 17	98 ± 18	.155
LAPWI area, cm^2^	11 ± 4	11 ± 4	10 ± 2	.169
Total radiofrequency time, min	66 ± 16	64 ± 15	74 ± 17	.079
Radiofrequency time for LAPWI, min	8 ± 5	8 ± 5	7 ± 4	.516
Low voltage area in the left atrium, cm^2^	57 ± 21	57 ± 22	56 ± 16	.936
Low voltage area in the posterior wall, cm^2^	10 ± 5	10 ± 5	9 ± 5	.513
CFAE area in the left atrium, cm^2^	31 ± 17	33 ± 17	23 ± 15	.130
CFAE area in the posterior wall, cm^2^	2 ± 3	2 ± 3	2 ± 2	.608

Note: Data are mean ± SD.

Abbreviations: AF, atrial fibrillation; CFAE, complex fractionated atrial electrogram; LAPWI, left atrial posterior wall isolation.

EAT overlap data are shown in Table 3. EAT overlaps on PVI lines, roof line, floor line, and mitral isthmus line were 0.1 ± 0.3, 1.0 ± 0.9, 0.0 ± 0.1, and 1.0 ± 0.8 cm^2^ respectively. Moreover, EAT overlaps on LAPWI lines and LAPWI area were 1.2 ± 1.0 and 0.5 ± 0.9 cm^2^ respectively. No significant correlations were found between the EAT overlap on the LAPWI area and the low voltage area or the CFAE area on the posterior wall (low voltage area, *R* = 0.222, *P* = .152; CFAE, *R* = 0.051, *P* = .744).

**Table 3:** Epicardial adipose tissue (EAT) overlaps on ablation lines and the left atrial posterior wall isolation (LAPWI) area

	All patients (n = 44)	AF‐free group (n = 34)	AF‐recurrent group (n = 10)	*P* value
EAT overlap on PVI lines, cm^2^	0.1 ± 0.3	0.2 ± 0.3	0.1 ± 0.2	.556
EAT overlap on the roof line, cm^2^	1.0 ± 0.9	1.2 ± 1.0	0.5 ± 0.5	.029
EAT overlap on the floor line, cm^2^	0.0 ± 0.1	0.1 ± 0.2	0.0 ± 0.0	.367
EAT overlap on the mitral isthmus line, cm^2^	1.0 ± 0.8	1.0 ± 0.8	1.0 ± 0.8	.952
EAT overlap on LAPWI lines, cm^2^	1.2 ± 1.0	1.4 ± 1.0	0.6 ± 0.6	.014
EAT overlap on LAPWI area, cm^2^	0.5 ± 0.9	0.6 ± 1.0	0.1 ± 0.3	.122

Note: Data are mean ± SD.

Abbreviations: AF, atrial fibrillation; CFAE, complex fractionated atrial electrogram; PVI, pulmonary vein isolation.

### 3.2 Outcome of catheter ablation and EAT

AF recurred in 10 patients (23%) during 21 ± 7 months of follow‐up. Accordingly, the study subjects were divided into an AF‐free group (n = 34 patients) and an AF‐recurrent group (n = 10 patients).

No significant differences were found between groups regarding the patients’ characteristics, including the rate of administration of antiarrhythmic drugs after catheter ablation, EAT volume (Table 1). Regarding catheter ablation data, the left atrial surface area and LAPWI area were not different between groups (Table 2). Moreover, although the total radiofrequency time tended to be shorter in the AF‐free group, no difference was found between groups regarding the radiofrequency time for LAPWI. Furthermore, no differences between groups were found in the low voltage and CFAE areas.

No significant differences were found between groups regarding EAT overlaps on PVI lines, floor line, and mitral isthmus line (Table 3); however, EAT overlap on the roof line was significantly larger in the AF‐free group than in the AF‐recurrent group (1.2 ± 1.0 vs 0.5 ± 0.5 cm^2^, *P* = .029). Moreover, although the EAT overlap on LAPWI area was not different between groups, the EAT overlap on LAPWI lines was significantly larger in the AF‐free group than in the AF‐recurrent group (1.4 ± 1.0 vs 0.6 ± 0.6 cm^2^, *P* = .014).

Univariate Cox regression analysis identified the left ventricular ejection fraction, total radiofrequency application time, and EAT overlap on LAPWI lines as factors for multivariate analysis (Table 4). Consequently, EAT overlap on LAPWI lines was the independent predictor of AF recurrence after catheter ablation (hazard ratio: 0.399, 95% confidence interval: 0.178‐0.891, *P* = .025).

**Table 4:** Univariate and multivariate Cox regression analysis for recurrence of atrial fibrillation (AF)

	Univariate	*P* value	Multivariate	*P* value
	Hazard ratio (95% CI)		Hazard ratio (95% CI)	
Age, years	0.975 (0.922 to 1.032)	.388		
Male gender	2.670 (0.690 to 10.335)	.155		
Body mass index, kg/m^2^	0.937 (0.758 to 1.159)	.549		
AF duration, months	0.988 (0.952 to 1.027)	.550		
Antiarrhythmic drugs after ablation	0.641 (0.181 to 2.272)	.491		
Left atrial volume, cm^2^	0.991 (0.973 to 1.009)	.302		
Left atrial appendage flow velocity, cm/s	0.992 (0.959 to 1.026)	.645		
Left ventricular ejection fraction, %	0.933 (0.860 to 1.013)	.099	0.932 (0.866 to 1.003)	.060
Total EAT volume, cm^3^	0.993 (0.975 to 1.010)	.314		
Left atrial EAT volume, cm^3^	1.000 (0.950 to 1.052)	.990		
LAPWI area, cm^2^	0.846 (0.682 to 1.049)	.127		
Total radiofrequency application time, min	1.036 (0.995 to 1.079)	.090	1.038 (0.994 to 1.084)	.093
CFAE area in the posterior wall, cm^2^	0.915 (0.683 to 1.226)	.553		
Low voltage area in the posterior wall, cm^2^	0.948 (0.822 to 1.094)	.466		
EAT overlap on LAPWI lines, cm^2^	0.370 (0.151 to 0.904)	.012	0.399 (0.178 to 0.891)	.025
EAT overlap on LAPWI area, cm^2^	0.224 (0.024 to 2.086)	.189		

Abbreviations: CFAE, complex fractionated atrial electrogram; EAT, epicardial adipose tissue; LAPWI, left atrial posterior wall isolation.

**Figure 2:**
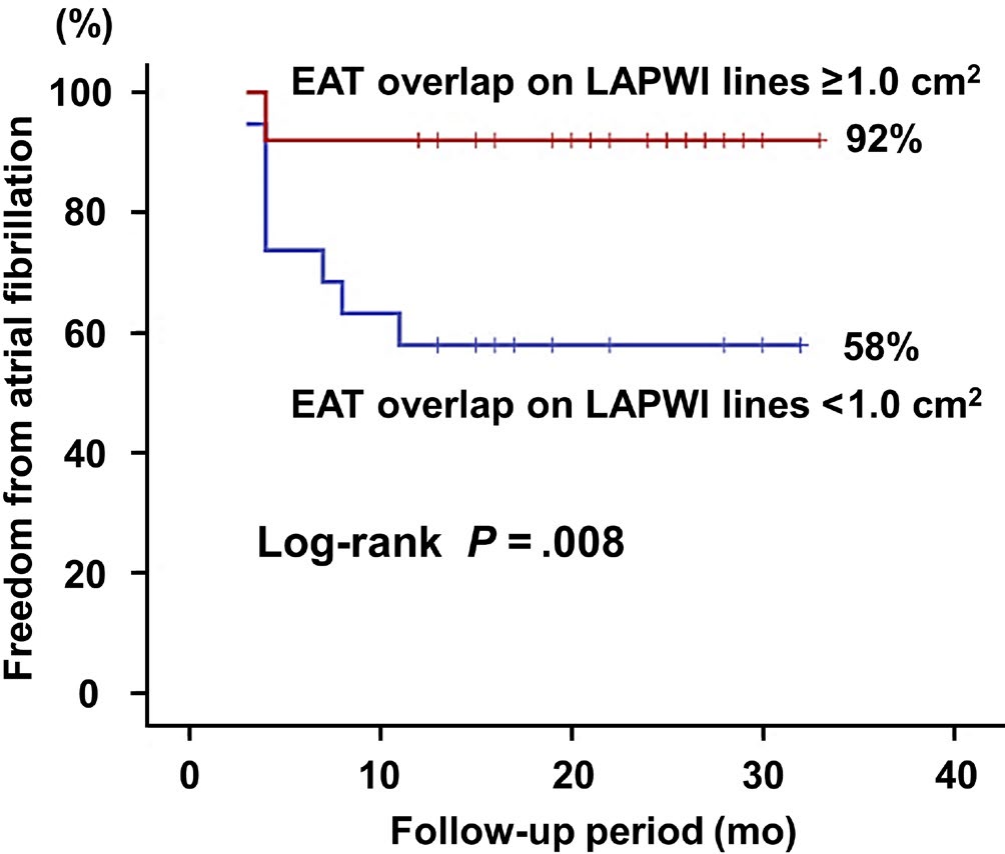
Kaplan‐Meier curves for atrial fibrillation (AF)‐free survival for large (≥1.0 cm^2^) or small (<1.0 cm^2^) epicardial adipose tissue (EAT) overlap on the left atrial posterior wall isolation (LAPWI) lines. The cut‐off value was determined by receiver operating characteristic curve analysis

In the receiver operating characteristic curve analysis, the EAT overlap on LAPWI lines yielded an area under the curve of 0.756 for prediction of AF recurrence (sensitivity, 80%; specificity, 68%; positive and negative predictive values, 43% and 92%, respectively, for a cut‐off value of 1.0 cm^2^). The difference between groups in AF‐free survival stratified by EAT overlap on LAPWI lines cut‐off of 1.0 cm^2^ was assessed using Kaplan‐Meier analysis (Figure 2), and demonstrated that 92% of the large EAT overlap group (≥1.0 cm^2^, n = 25) remained AF free compared to only 58% of the small EAT overlap group (<1.0 cm^2^, n = 19) during follow‐up (*P* = .008).

## 4 DISCUSSION

The present study evaluated the impact of EAT on AF recurrence after catheter ablation with LAPWI for persistent AF patients. The study's main findings were as follows: (a) EAT volume was not associated with AF recurrence after catheter ablation; (b) EAT overlap on the LAPWI area did not correlate with low voltage area or with CFAE area on the posterior wall; (c) Although EAT overlap on the LAPWI area was not related to AF recurrence, large EAT overlap on LAPWI ablation lines was associated with a high AF freedom rate after catheter ablation.

The local effect of EAT is partly demonstrated by the direct adipocyte infiltration into the underlying atrial myocardium. A previous experimental study using an ovine model revealed that obesity caused EAT accumulation with pronounced myocardial infiltration by adipocytes, particularly over the left atrial posterior wall.^9^ Such direct fatty infiltration separating myocytes could lead to conduction slowing and heterogeneity.^20^, ^21^ Moreover, the fact that profibrotic adipokines secreted from EAT facilitate paracrine effects on the atrial myocardium is another possible mechanism of the local effect. A previous study^12^ reported that EAT secretome promotes myocardial fibrosis through secretion of fibrotic adipokines, such as Activin A. In fact, previous studies^7^, ^8^ have reported that EAT distribution is related to the location of low voltage areas and fractionated potentials. In contrast, the present study showed that EAT overlap on the LAPWI area is not correlated with the low voltage area or with the CFAE area on the left atrial posterior wall. Therefore, the local effect of EAT may not have primarily contributed to generate AF substrate.

EAT also exerts a remote effect on the atrial myocardium, which arises from the systemic secretion of adipokines and metabolites. For instance, increased leptin levels can lead to increased aldosterone secretion, endothelial dysfunction, increased vascular stiffness, hypertension, and cardiac hypertrophy, all of which could contribute for AF pathogenesis.^22^ Moreover, adipokines and metabolites from EAT are associated with systemic inflammation, oxidative stress, and autonomic dysfunction, resulting in a pro‐arrhythmogenic state.^14^

Another potential mechanism underlying the remote effect involves the fact that EAT contains ganglion plexuses, which play a critical role for AF initiation and perpetuation.^23^ Increased ganglion plexus activity induces AF through shortening action potential duration via parasympathetic stimulation and an increased calcium loading via sympathetic stimulation.^23^ In the present study, direct application of radiofrequency to the area overlapped with EAT may have suppressed AF recurrence through modification of ganglion plexuses. This hypothesis is supported by previous studies that revealed the AF‐suppressing effect of radiofrequency application targeting ganglion plexuses.^23^, ^24^ Furthermore, the finding of the present study that EAT overlap on the mitral isthmus line was not associated with AF recurrence is consistent with this hypothesis. Because the major ganglion plexuses are located around the pulmonary vein antrum and the left atrial posterior wall.^23^, ^24^

The findings of the present study suggest that the AF‐promoting effect of EAT is mediated mainly by the remote effect. If the local effect comprised the main pathway underlying EAT promoting AF recurrence, then EAT overlap on the LAPWI area should be related to AF recurrence after ablation. Previous studies reported the effectiveness of catheter ablation to target areas overlapped with EAT.^17^, ^18^ Applying radiofrequency to the high dominant frequency sites overlapped with EAT suppresses AF recurrence after catheter ablation.^17^ Moreover, EAT‐based ablation achieved relatively high AF freedom rate compared with generalized stepwise ablation.^18^ Although the effectiveness of the direct radiofrequency application to the areas overlapped with EAT was revealed in these studies,^17^, ^18^ the effectiveness of isolating EAT overlapped area remains unclear. Therefore, the present study is the first to suggest the remote effect as a primary cause of the AF‐promoting effect of EAT.

### 4.1 Clinical implications

The results from the present study show that creating LAPWI lines to cross the EAT overlap area may be critical to increase the effectiveness. Moreover, the occurrence of EAT overlap areas within LAPWI may imply that additional radiofrequency applications directly to those areas may effectively suppress AF. Previous studies have shown an association between EAT volume and AF recurrence after PVI.^4^, ^5^ In contrast, no such association was found in the present study and this discrepancy may be due to LAPWI reducing the impact of EAT overlap on AF recurrence. This hypothesis is supported by a previous study reporting no contribution of EAT volume for AF recurrence after LAPWI with epicardial ablation.^25^ Therefore, LAPWI may be effective, especially for patients with a large EAT volume.

### 4.2 Study limitations

The present study was limited in several ways. First, the number of patients was too small to draw definite conclusions. Second, the present study included only patients who underwent LAPWI. Thus, it is unclear whether the impact of EAT overlap on AF recurrence can be observed in other ablation strategies. Third, we used a decapolar circular catheter (Libero) for the voltage and CFAE mapping, whereas using a dedicated mapping catheter with a short interelectrode distance could have allowed more detailed recordings of local electrograms. Fourth, AF recurrence was evaluated using surface electrocardiograms and 24‐h Holter monitoring. Accordingly, asymptomatic AF recurrences may have been overlooked in the present study. An implantable loop‐recorder may reveal a more accurate AF‐recurrence rate. Fifth, although previous studies revealed that a radiofrequency application from atrial endocardium modified the ganglion plexuses,^23^, ^24^ alteration in parasympathetic nerve activity was not assessed in the present study. Finally, it is unclear whether radiofrequency application to the area overlapped with EAT can suppress the secretion of adipokines and metabolites from EAT. Therefore, the detailed mechanism of the AF‐suppressing effect of radiofrequency application to EAT overlap remains unclear. Further studies are required to assess the relationship between changes in the serum adipokine levels and EAT overlap on ablation sites.

## 5 CONCLUSION

Although EAT overlap on LAPWI did not relate to AF recurrence, large EAT overlap on the LAPWI ablation lines was associated with high AF freedom rate after catheter ablation. This suggests that the AF‐promoting effect of EAT is more mediated by the remote effect than by the local effect. Therefore, direct radiofrequency application to EAT overlap may be necessary for AF suppression.

## CONFLICT OF INTEREST

Authors declare no Conflict of Interests for this article.
